# An Autopsy Case of Idiopathic Rhabdomyolysis in 1979: Immunoperoxidase Detection of Myoglobin Casts in Formalin-Fixed, Paraffin-Embedded Sections of the Kidney

**DOI:** 10.7759/cureus.18922

**Published:** 2021-10-20

**Authors:** Yutaka Tsutsumi

**Affiliations:** 1 Diagnostic Pathology Clinic, Pathos Tsutsumi, Inazawa, JPN; 2 Medical Technology, Yokkaichi Nursing and Medical Care University, Yokkaichi, JPN

**Keywords:** historical comments, autobiographical case report, rhabdomyosarcoma, myoglobinuria, myoglobin casts, immunoperoxidase method, idiopathic rhabdomyolysis, formalin-fixed paraffin-embedded sections, diagnostic pathology

## Abstract

In 1979, the author in his younger days experienced an autobiographical case of idiopathic rhabdomyolysis. The heme casts in formalin-fixed, paraffin-embedded sections of the kidney were immunoreactive for myoglobin. In these days, the immunoperoxidase technique had been utilized as a research seed by using paraformaldehyde-fixed frozen sections. The precious experience prompted the young author of his younger days to apply the immunoperoxidase method to diagnostic pathology using formalin-fixed paraffin-embedded sections. A brief history of early development of chromogenic immunostaining in diagnostic pathology in Japan is summarized.

## Introduction

In the summer in 1979, the author aged at 27 years as a third-grade postgraduate student met a Danish man on an autopsy table in the pathology department. The male patient died of acute rhabdomyolysis of unknown etiology. His onset was in an aircraft. The autopsied formalin-fixed, paraffin-embedded (FFPE) kidney contained heme or granular casts, which were clearly immunoreactive for myoglobin. At that time, it was never popular for us pathologists to immunostain antigens in FFPE sections. Chromogenic immunostaining (immunoperoxidase technique) had commonly been utilized as a research seed by using paraformaldehyde-fixed frozen sections. A brief case report was described in a Japanese journal [1].

This successful experience prompted the young author to apply immunostaining to diagnostic pathology using FFPE sections. Actually, rhabdomyosarcoma cells with eosinophilic cytoplasm were positively immunostained for myoglobin. This might be the very beginning of immunoperoxidase-assisted diagnostic pathology in Japan, the author believes so. The author dares to describe herein an epoch-making personal experience as his autobiographical case report.

## Case presentation

Clinical summary

A 43-year-old Danish man who had suffered from persistent occipito-nuchal pain for 10 years flew to Japan for sightseeing in June, 1979. He vomited in the airplane and complained of sever malaise. Next day, progressive muscle weakness and dyspnea on exertion appeared. On day 3, myalgia of both thighs started, and marked sweating happened on day 4. He was admitted to a local hospital in Tokyo on day 5. Urine excretion was kept, but the urine color was black. He smoked cigarettes and drank alcohol, but did not use any special medication. No family history of muscle disease was recorded. On admission, his mental status was clear. Hypothermia (35°C), tachycardia (120/minutes), and hypertension (150-170/100-120 mmHg) were pointed out. He complained of cold sensation on the lower extremities. Because of systemic myalgia with tenderness, he could not sit up on the bed. Decrease of urine volume (415 mL/day) with black-colored appearance persisted. The laboratory data on admission included neutrophilia (20,000/µL), blood urea nitrogen 42 mg/dL, creatinine 2.0 mg/dL, aspartate transaminase 700 IU/L, alanine aminotransferase 130 IU/L, lactate dehydrogenase 1,860 IU/L, sodium 137 mEq/L, potassium 5.4 mEq/L, chloride 95 mEq/L, and calcium 3.7 mEq/L. The urine revealed macroscopic hematuria but without red cells in the sediment. Myoglobin was immunologically detected in his urine. On day 7, he expired suddenly and unexpectedly. No dialysis therapy was given throughout his illness.

Autopsy findings

**Figure 1:**
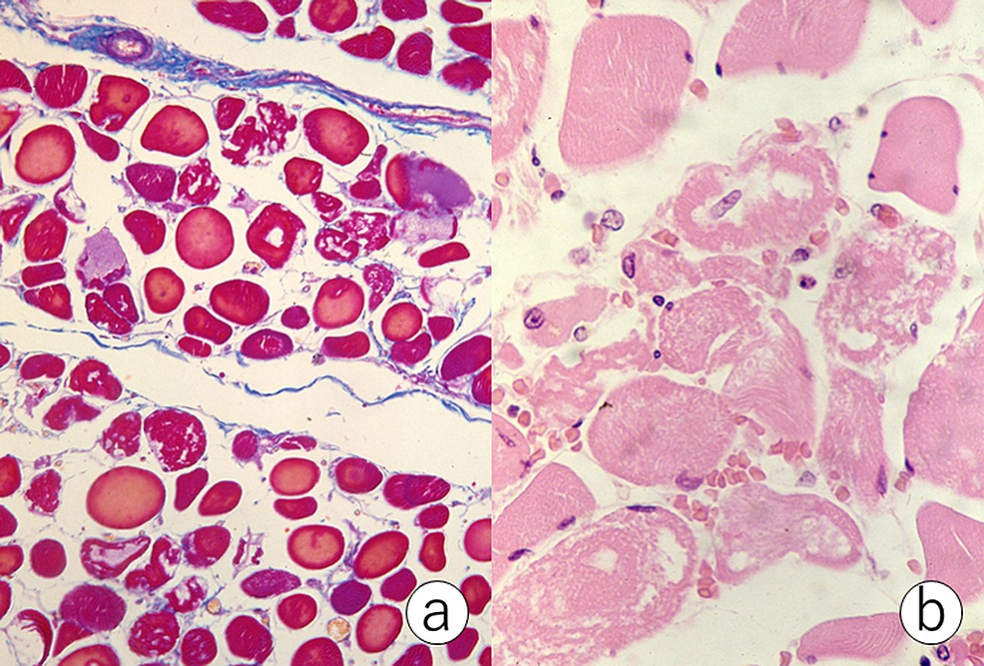
Rhabdomyolysis (a: Azan-Mallory, b: hematoxylin and eosin). Myolytic fibers are microscopically evident in the striated muscle. Inflammatory cellular reactions are minimal.

Autopsy was performed 6 hours after death in the Department of Pathology, Keio University School of Medicine, Tokyo, Japan. A well-muscled body weighed 72 kg, with height of 175 cm. No gross abnormality was discerned in skeletal and cardiac muscles (Figure 1).

**Figure 2:**
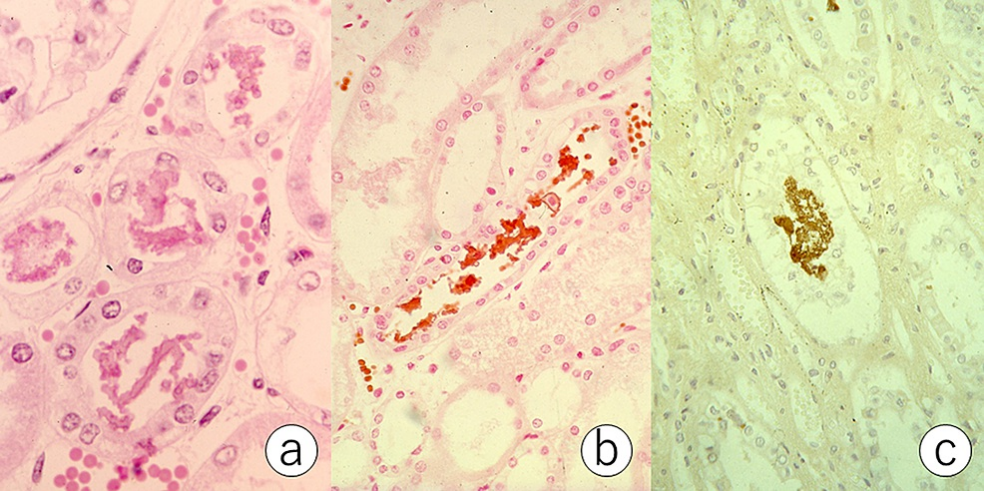
Myoglobin casts in the lower nephron (a: hematoxylin and eosin, b: Ralph’s benzidine reaction, c: PAP stain for myoglobin). Eosinophilic granular casts are seen in the lumen of the distal convoluted tubules. Red blood cells and the casts are positive with Ralph’s benzidine reaction, and the casts are immunoreactive for myoglobin. PAP, peroxidase-antiperoxidase.

Microscopically, myolytic fibers were dispersed in both skeletal and cardiac muscles. Cellular reactions were sparse: infiltration of macrophages and neutrophils was minimal. The kidneys weighed 160 g (left) and 140 g (right). Eosinophilic granular casts were observed in the lower nephrons, and the casts were positive for Ralph’s benzidine reaction [2] (Figure 2a, 2b).

The casts in the FFPE sections showed myoglobin immunoreactivity with a peroxidase-antiperoxidase (PAP) method [3] (Figure 2c). The direct cause of death was pulmonary edema due to acute heart failure. The cause for his persistent nuchal pain was not clarified.

Liquid chromatography tests for arsenics and heavy metals were negative in the liver. Viral isolation trials using the -20°C frozen striated muscles, kidney, and serum were unsuccessful: in vitro experiment with human erythroleukemia cells and in vivo experiment with suckling mice failed to identify any virus. Immunofluorescence studies for Coxsackie viruses (A12, B3, B4, and B5) and herpes simplex virus were negative.

The final anatomical diagnosis was idiopathic rhabdomyolysis with acute renal failure due to myoglobin cast formation.

## Discussion

Rhabdomyolysis was caused by a variety of factors, including hereditary muscle disorders, exertions/convulsions, traumas, metabolic disorders, myositis, hypokalemia, toxins, and drugs [4]. Drugs against hyperlipidemia such as fenofibrate and statins may provoke rhabdomyolysis, and neuroleptics may cause neuroleptic malignant syndrome. In the present case, no particular cause for rhabdomyolysis was identified, hence the diagnosis of idiopathic rhabdomyolysis.

It is known that patients with sickle cell disease or sickle cell trait of hemoglobin S may manifest acute hypoxic attack in an unpressurized aircraft [5]. The Danish man first manifested in an airplane, so that the possibility of “myoglobinopathy” was considered. A recently reported disease entity myoglobinopathy shows progressive chronic myopathy with an autosomal-dominant trait [6], but an acute disease as seen in the present case has not been described.

A consultant professor of renal pathology in the department asked me “Do you have frozen sections or alcohol-fixed material?” My answer was “No, I don’t have them”. The professor said “Then, it’s impossible for you to identify myoglobin in FFPE sections. You’d better give up.” However, I knew that anti-myoglobin rabbit antiserum was commercially available, and a PAP kit supplied from DAKO company (Copenhagen, Denmark) was kept in a refrigerator in the department. It was well known that peptide hormones were reproducibly detectable in FFPE sections [7], and some reports described immunoglobulin immunoreactivity in FFPE sections [8]. These were the reasons why the author dared to try to demonstrate myoglobin in the FFPE sections of the kidney. The patient’s striated muscle tissue was used as a positive control. Then, I realized that the advice of an experienced specialist was not almighty. Actually, the heme casts in the distal convoluted tubules, as well as the cytoplasm of the striated muscle cells, clearly showed distinct brown signals.

At that time, the Department of Pathology, Keio University School of Medicine, regularly held surgical pathology conferences, principally based on the findings of hematoxylin and eosin-staining. Soft tissue sarcomas often became a target of intense discussion. The diagnosis of rhabdomyosarcoma was especially difficult. We should look for cross striations in phosphotungstic acid-hematoxylin-stained sections, but it was time-consuming and often inaccurate.

**Figure 3:**
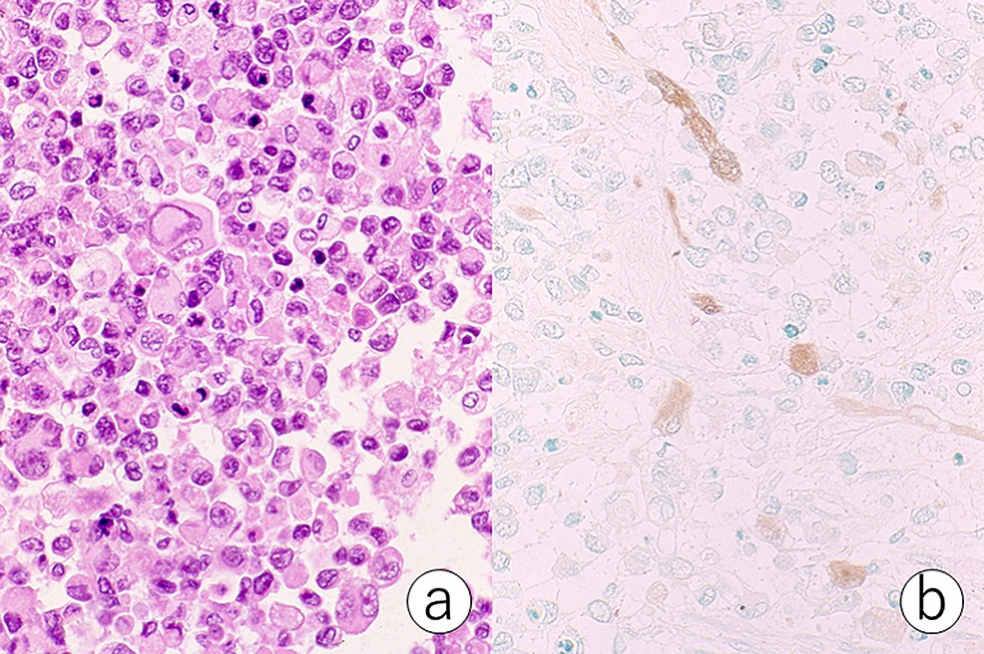
Rhabdomyosarcoma of prostate origin metastatic to the lung (a: hematoxylin and eosin, b: PAP stain for myoglobin). Small round cells with pleomorphic nuclei and eosinophilic cytoplasm are noted. Some of the tumor cells are immunoreactive for myoglobin. PAP, peroxidase-antiperoxidase.

I applied PAP staining of myoglobin to the diagnosis of rhabdomyosarcoma. Brown signals were easily recognized in some atypical spindle cells, particularly with eosinophilic cytoplasm, in FFPE sections (Figure 3).

It happened in a fall of 1979. This might be the very beginning of immunoperoxidase-assisted diagnostic pathology in Japan, the author believes so.

In May, 1980, I moved to the Department of Pathology, Tokai University School of Medicine, Isehara, Kanagawa, Japan. Prof. Keiichi Watanabe (Department of Pathology) and Prof. Paul K. Nakane (Department of Cell Biology) of the university were the co-leaders of immunohistochemistry in Japan. Prof. Nakane was the founder of the immunoperoxidase technique [9]. Many Japanese investigators came up to learn immunohistochemistry in annual training courses held by them. Under the warm support by the two professors, I started to establish a novel diagnostic system for immunoperoxidase application to diagnostic pathology in Tokai University Hospital. The author of his younger days evaluated numbers of primary antibodies applicable to FFPE sections, and the diagnostic system was transported to two city hospitals. In early 1980s, the author was frequently invited to give lectures on the application to diagnostic pathology and noteworthy pitfalls and caveats of the method throughout Japan. In 1984, the author summarized a series of review articles for immunoperoxidase application to diagnostic pathology in Japanese [10-13], and in 1985, the detailed content was described in a Japanese-written textbook entitled the enzyme-labeled antibody method edited by Prof. Keiichi Watanabe and Prof. Paul K. Nakane [14]. The fourth edition of the textbook was published in 2002 as a long-seller textbook [15]. Recently, a review article on the pitfalls and caveats in diagnostic immunohistochemistry was published [16].

In the starting period, it was harsh for us to obtain antibodies applicable to FFPE sections. An original menu for immunostaining markers was limited to antisera to immunoglobulins (IgG, IgA, IgM, kappa, lambda and J-chain), tumor markers (carcinoembryonic antigen [CEA], alpha-fetoprotein, and human chorionic gonadotropin), lysozyme, secretory component, epithelial membrane antigen (EMA), S-100 protein, factor VIII-related antigen, neuron-specific enolase (NSE), glial fibrillary acidic protein, thyroglobulin, Bacille Calmette Guérin antigen (to detect Mycobacterial antigens by a rabbit antiserum), human papillomavirus, hepatitis B surface (HBs) antigen, and hepatitis B core (HBc) antigen. In 1982, the author and a colleague encountered an impressive case of amelanotic melanoma massively metastatic to an axillary lymph node, in which immunostaining for S-100 protein and NSE significantly contributed to the final histopathological diagnosis [17].

In 1975, Georges Köhler and César Milstein first succeeded in making monoclonal antibodies by creating hybridomas. In early 1980s, several monoclonal antibodies, including leukocyte common antigen (LCA; CD45) and Leu 7 (CD57), became commercially available [18]. Thereafter, cytokeratin subtypes, desmin, vimentin, smooth muscle actin, CEA antigen (not reactive with nonspecific cross-reacting antigen), and lymphocyte surface markers were stably detectable in FFPE sections. The author demonstrated that a mouse monoclonal antibody KL-1 to cytokeratin was useful to consistently detect columnar epithelial cells and adenocarcinoma cells, and mouse monoclonal antibodies MB-1 and MT-1 were applicable to identifying B and T cells in FFPE sections. By presenting at seminars and in publications spoken and written in Japanese, the author tried to disseminate these monoclonals throughout Japan to apply to the immunohistochemical diagnosis. In 1984, immunostaining for diagnostic pathology became to be partly covered with the public payment system for medical services, which significantly accelerated this application. Questionnaire survey indicated that by 1986, half of 500 Japanese city hospitals had employed chromogenic immunostaining for routine diagnostic pathology [19].

Table 1 summarizes immunohistochemical markers frequently used for diagnostic pathology in the period 1984 through 1988 in the division of diagnostic pathology of Tokai University Hospital, Isehara, Japan [19].

**Table 1:** Immunohistochemical markers commonly used for diagnostic pathology in the period 1984 through 1988 in the division of diagnostic pathology of Tokai University Hospital CEA, carcinoembryonic antigen; AFP, alpha-fetoprotein; HCG, human chorionic gonadotropin; EMA, epithelial membrane antigen; NSE, neuron-specific enolase; GFAP, glial fibrillary acidic protein; BCG, Bacille Calmette Guérin; HPV, human papillomavirus; HBs, hepatitis B surface; HBc, hepatitis B core; LCA, leukocyte common antigen

Marker	1984	1985	1986	1987	1988	Total
Keratin (KL-1)	96	147	181	248	207	879
CEA	100	157	145	173	158	733
LCA	42	63	134	133	116	488
Vimentin	45	70	101	130	85	431
S-100 protein	55	86	104	101	77	423
EMA	48	98	83	101	55	385
HBs antigen	35	53	65	92	99	344
T-cell (MT1, UCHL1)	-	33	89	100	117	339
B-cell (MB1, L26)	-	32	85	96	119	332
Lambda chain	50	60	74	71	71	326
Kappa chain	49	60	75	70	71	325
HBc antigen	23	47	64	88	94	316
IgG	45	50	60	80	64	299
IgM	47	48	61	77	62	295
IgA	37	47	58	76	58	276
Desmin	17	47	59	65	46	234
AFP	28	60	46	53	42	229
BCG	45	61	46	47	30	229
Leu M1 (CD15)	-	8	72	77	65	222
NSE	35	31	38	55	42	201
Lysozyme	18	25	52	49	50	194
CA19-9	-	28	44	49	68	189
Calcitonin	3	19	45	46	37	150
J-chain	33	29	32	28	27	149
Chromogranin A	-	21	49	46	29	145
Secretory component	22	21	38	26	30	137
GFAP	21	21	35	27	27	131
Factor VIII-related antigen	14	10	42	39	23	128
Thyroglobulin	3	22	31	34	31	121
HCG	12	41	27	27	13	120
HPV	3	13	34	45	23	118
Myoglobin	12	22	35	33	13	115
Keratin (antiserum)	-	-	-	78	28	106
Neurofilament	6	11	37	32	17	103

Among some 150 immunohistochemical markers, the top 10 included keratin (KL-1), CEA, LCA, vimentin, S-100 protein, EMA, HBs antigen, T-cell markers (MT-1 and UCHL1), B-cell markers (MB-1 and L26), and lambda and kappa chains of immunoglobulins. Myoglobin was ranked at the 27th position of the markers used.

In 1991, Shi et al. invented a miraculous step of hydrating heating to retrieve the antigenicity in FFPE sections [20]. Heating of deparaffinized sections in a variety of solutions using a microwave or autoclave has considerably contributed to the increase of detection sensitivity. The development of high-sensitivity detection systems such as avidin-biotinylated peroxidase method, polymer techniques, and catalyzed signal amplification method also consists of an important aspect in the consistent use of immunostaining for diagnostic pathology.

There is no solid principle of which antigens are suitable for staining on FFPE sections. Generally speaking, antisera may better stain antigens in FFPE sections than monoclonals. The information from the manufacturer will help us immunostain antigens. Anyway, we should try to immunostain the antigens using FFPE sections.

Nowadays, the immunoperoxidase technique is indispensable and essentially important for the appropriate pathology diagnosis, and so many polyclonal and monoclonal antibodies are available from commercial sources. We have reached considerable technical development, as described above. The application has been widespread throughout Japan, and is significantly contributory to the appropriate histopathological diagnosis in every hospital equipped with diagnostic pathology. When necessary, hospital diagnostic pathologists can easily access to the public or regional consultation system, in which specialists provide with their opinion commonly using immunohistochemical findings. As one of the pioneers, the recent development in this field is so moving to me. I cordially appreciate the Danish gentleman who gave the developing author an epoch-making personal experience.

## Conclusions

An autobiographical case of idiopathic rhabdomyolysis in 1979 was re-described. Immunoperoxidase method for myoglobin was applicable to FFPE sections of the kidney to identify the myoglobin casts in the distal convoluted tubules. In those days, the immunoperoxidase technique had been utilized as a research seed by using paraformaldehyde-fixed frozen sections. This valuable experience prompted the author of his younger days to apply the immunoperoxidase method to diagnostic pathology using FFPE sections. A brief note on the history of diagnostic immunohistochemistry in Japan was described.
